# Clinical findings and management of diaphragmatic rupture with hernia caused by safety body harness: A case report

**DOI:** 10.1016/j.amsu.2021.102429

**Published:** 2021-05-29

**Authors:** Adeodatus Yuda Handaya, Aditya Rifqi Fauzi, Joshua Andrew, Ahmad Shafa Hanif, Kevin Radinal, Azriel Farrel Kresna Aditya

**Affiliations:** Digestive Surgery Division, Department of Surgery, Faculty of Medicine, Universitas Gadjah Mada/Dr. Sardjito Hospital, 55281, Faculty of Medicine, Universitas Gadjah Mada/Dr Sardjito Hospital, Yogyakarta, Indonesia

**Keywords:** Diaphragmatic rupture, Traumatic diaphragm hernia, Laparotomy, Reinforced suture

## Abstract

**Introduction:**

Acute blunt traumatic diaphragmatic rupture (BTDR) caused by falling from a height is rare. Transabdominal diaphragmatic repair in an acute setting following BTDR requires good clinical decision-making and diagnostic tests.

**Case presentation:**

A 36-year-old male was involved in a work accident. He fell from a 30-m radio transmitter tower while wearing an attached safety body harness. He arrived in the emergency room with complaints of breathing difficulty, abdominal and pelvic pain. We discovered a diaphragmatic rupture with abdominal organ herniation based on the imaging. We decided to perform an emergency laparotomy. We discovered a 12cm diaphragmatic defect on the anteromedial side of the left during surgery. We carried out the evacuation by suction and controlled the bleeding in the wound at the edge of the diaphragm. On postoperative day 4 (POD), the patient complained of dyspnea, and chest radiology revealed a hemothorax in the left lung. We then installed a water-sealed drainage (WSD) until POD 6. On the following day, his complaint was resolved, the WSD was removed and the patient was discharged uneventfully.

**Discussion:**

Abdominal CT scan can be helpful in determining early diagnosis of traumatic diaphragm rupture with abdominal organ herniation, allowing for prompt surgical intervention to minimize morbidity and mortality. Furthermore, reinforced sutures might be useful to prevent recurrence of the symptoms.

**Conclusion:**

In conclusion, injury due to wearing a safety body harness when falling can be a potential cause of BTDR. Management of BTDR transabdominally is a safe and effective procedure.

## Highlights

• The preoperative diagnosis of TDH is still a challenge.
• Safety body harness can be a potential cause of blunt traumatic diaphragm rupture.
• CT scan chest is superior in diagnosing diaphragmatic rupture than x-ray.
• Suture technique might have an effect to prevent symptom recurrence.

## 1 Introduction

Blunt traumatic diaphragmatic rupture (BTDR) is a major complication of abdominal or thoracic trauma. BTDR and traumatic diaphragmatic hernia (TDH) appear more often in males than females in trauma patients, with a prevalence of 0.8%–5%. TDH is more common in blunt trauma than penetration injury (13.3 vs. 55%), and has been identified in unusual cases of iatrogenic trauma such as chest tube penetration. In blunt trauma, it is more prominent on the left side and has a greater diameter and dimension [1]. Diaphragmatic injuries are difficult to identify and are often delayed, resulting in increased morbidity and mortality [2]. This report is a review of our experience with traumatic diaphragmatic rupture in a male who fell from a radio transmitter tower while wearing an attached safety body harness. This work has been reported in line with the SCARE checklist [3].

## 2 Presentation of case

**Fig. 1:**
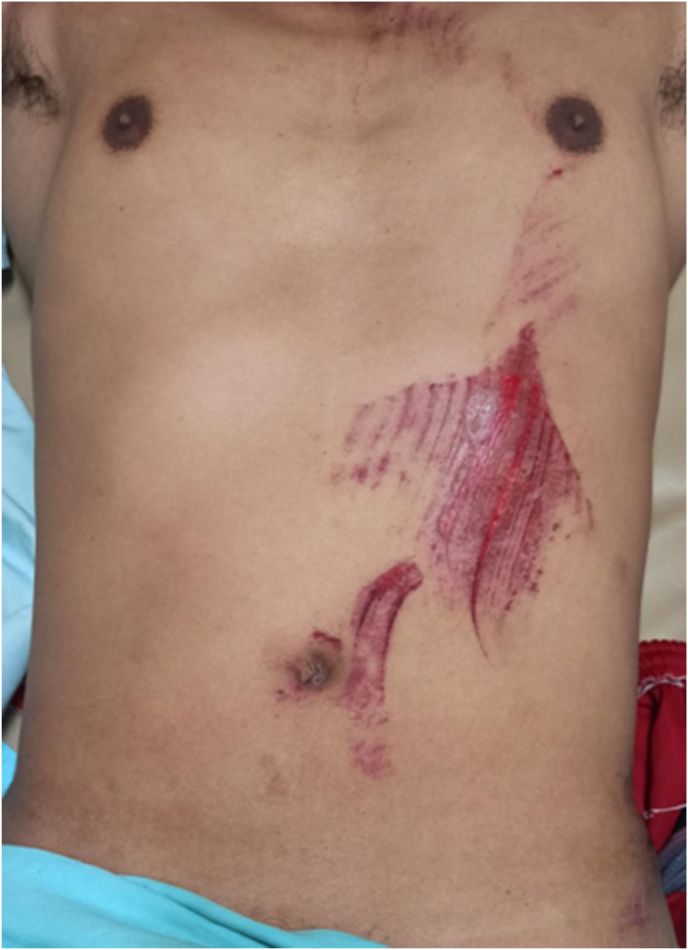
Patient presentation in emergency room, showing blunt abdominal injury.

**Fig. 2:**
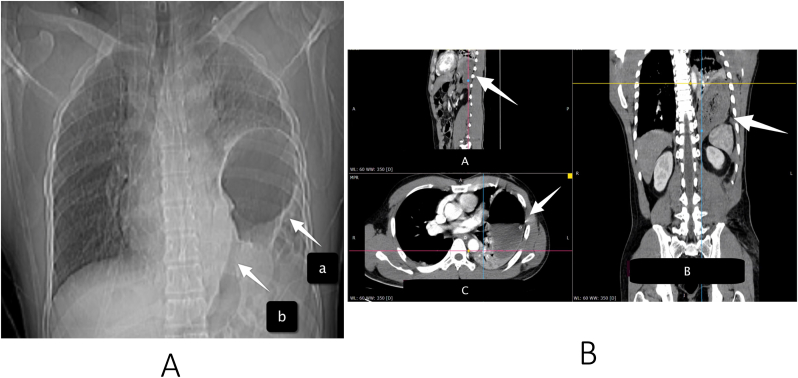
Left: Patient's initial chest x-ray, air sac (a) in the left thorax associated with the abdominal cavity (b); Right: A) Abdominal CT Scan-sagittal view B) coronal view C) axial view. The arrow shows the hollow viscus organ entering the thoracic cavity.

**Fig. 3:**
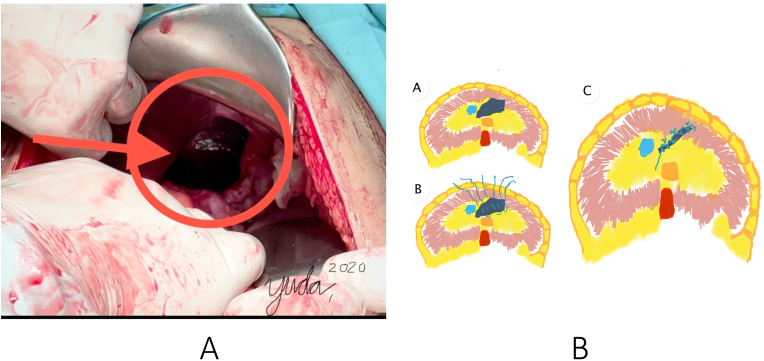
a) diaphragmatic defect on the anteromedial side of the left with a diameter of 12cm; b) schematic illustration of the diaphragm repair with horizontal mattress sutures reinforced with simple continuous sutures using nonabsorbable thread.

A 36-year-old male was involved in a work accident. He fell from a 30-m radio transmitter tower with a safety body harness attached, and hit a house roof 12-m tall. He arrived in the emergency room with complaints of breathing difficulty, abdominal and pelvic pain (Fig. 1). He was managed according to Advanced Trauma Life Support's primary and secondary survey protocol in the nearest rural hospital. He received wound control for the abdominal wall and pelvic wound, and then was referred to our trauma centerin a referral hospital in Yogyakarta, Indonesia for his multiple traumas: maxillofacial and pelvic injuries. No relevant significant past medical history was found on the patient. On initial physical examination, bowel sounds were heard in the left hemithorax area. Then, he was sent for initial chest radiography and it demonstrated air sac (a) in the left thorax associated with the abdominal cavity (b) consistent with left diaphragmatic hernia (Fig. 2a). We performed focused assessment sonography on trauma (FAST) and we found minimal free fluid in the splenorenal. An abdominal computed tomography (CT) scan was performed to find out if there was any other injury to the abdomen or not. The findings revealed the presence of a portion of his stomach and small intestines in the left hemithorax, as well as traumatic diaphragmatic rupture but no solid organ rupture (Fig. 2b). He underwent an emergency exploratory laparotomy by a senior gastrointestinal surgeon, and we found hematoma in the left pericolic region. The solid organ was within normal limits, and a minimum hemoperitoneum of about 100 cc was found. We discovered that the gastric and transverse colons had herniated into the diaphragm. The stomach and colon were then extracted and evacuated into the abdominal cavity. Intraoperatively, we discovered a diaphragmatic defect on the anteromedial side of the left with a diameter of 12cm and a pleural hemorrhage of approximately 50 cc (Fig. 3a). We carried out the evacuation by suction and controlled the bleeding in the wound at the edge of the diaphragm. Then, as shown in Fig. 3b, we repaired the diaphragm with horizontal mattress sutures made of non-absorbable vicryl 0 multiple threads. The patient was then given maximal inspired air at the last mattress suture to drain blood from the pleural cavity. Sutures were reinforced with simple non-absorbable continuous sutures made of vicryl 0 thread.

On postoperative day 2, the patient had no complaints. We performed chest x-ray evaluation on POD 3 and the result revealed lung opacity was increased in left hemithorax. On POD 4, the patient complained about dyspnea, and a chest radiology evaluation showed hemothorax in the left lung. We then installed water-sealed drainage (WSD) on his left hemithorax. The initial fluid that came out was 100 cc of blood. On POD 6, the WSD was removed, and the next day the patient was discharged uneventfully. Follow-up in the outpatient clinic on the 7th and 14th days after discharge revealed that the patient's wound was healing well and that his symptoms had not returned.

## 3 Discussion

Traumatic diaphragmatic hernia (TDH) is an exceptionally rare and often life-threatening injury caused by blunt or penetrating trauma. Today, the rate of falls is growing every day as the number of high-rise buildings grows. The preoperative diagnosis of TDH is still a challenge. Diagnosing diaphragmatic rupture after blunt thoracoabdominal trauma in acute settings requires a high index of suspicion [4]. The level of TDH in the left hemidiaphragm is higher than in the right due to the lack of any protection from the compressing forces transmitted by blunt trauma [5], but ironically, our case was not without protection. He used a safety body harness. The left side accounts for about 65% of diaphragmatic ruptures [6]. This was similar to our case and might be caused by the sudden increase of intraabdominal pressure by the fall from a height. As a result, his abdomen compressed with the safety body harness, causing rupture of the diaphragm and pushing upward his abdominal organs.

When compared to traditional X-ray exams, a trauma CT scan chest is superior in diagnosing diaphragmatic rupture [7]. The presence of related injuries in the thoracic or abdominal, should be managed by emergency surgery. Hospital capability, and the surgeons' expertise are important to prevent the recurrence of diaphragmatic rupture repair. Thoracotomy or laparotomy should be used in the absence of thoracic or abdominal viscera trauma, depending on the surgeon's choice or experience and location of other suspected injuries [8,9]. The suture technique might also have an effect on symptom recurrence. Herniated abdominal organs were returned to the abdomen in our case, and the defect was successfully repaired with a laparotomy using a horizontal mattress and simple continuous sutures.

There has not been any evidence of diaphragmatic rupture healing on its own. Surgery is the next step for a patient with TDH after a diagnosis has been made [8], and is the most successful and safe therapy. TDH is treated with a variety of surgical techniques, including thoracotomy, laparotomy, and thoracotomy with laparotomy, video-assisted thoracic surgery, and laparoscopy. The most popular procedure for TDH is laparotomy, which involves a complete exploration of the abdominal viscera [9]. When there are no abdominal injuries, however, it is better to use a thoracotomy to reduce the herniated tissues and repair the diaphragm [10]. In our case, we decided to perform laparotomy because the patient had abdominal injuries. We found diaphragmatic rupture and pelvic injuries, and the patients underwent laparotomy successfully. The rupture of the diaphragm with other intraabdominal injuries was safer to be repaired via laparotomy. Another reason to choose the abdominal approach is that we could prevent misdiagnosis and reduce patients’ morbidity and mortality caused by intraabdominal injury.

The majority of the reports did not have recurrence rates after surgery [4]. The patient in our report was being followed up, and no recurrence has been observed. The suture process, in our view, is critical for recurrence rates. The diaphragmatic defect was corrected in our case using nonabsorbable horizontal mattress sutures reinforced by plain continuous nonabsorbable sutures. Mesh repair is needed when the defect is wide enough to require tension-free repair [11,12]. We decided to perform surgery and defect closure without using mesh because the defect could be approximated without tension, and the availability of mesh with anti-adhesive surface is usually not available in emergency settings.

In our patient, during postoperative care, dyspnea developed. The chest x-ray evaluation found minimal hemothorax, so we performed WSD insertion. We suggest that in the case of TDH with bleeding in the thoracic cavity, it is better to insert the WSD earlier postoperatively.

## 4 Conclusions

To summarize, TDH can be caused by an injury due to wearing a safety body harness. Management of TDH transabdominally is a safe and effective procedure.

## Patient consent

The informed consent form was declared that patient data or samples will be used for educational or research purposes. Our institutional review board also do not provide an ethical approval in the form of case report.

## Sources of funding

The authors declare that this study had no funding source.

## Ethical Approval

The informed consent form was declared that patient data or samples will be used for educational or research purposes. Our institutional review board also do not provide an ethical approval in the form of case report.

## Consent

Written informed consent was obtained from the patient for publication of this case report and accompanying images. A copy of the written consent is available for review by the Editor-in-Chief of this journal on request.

## Author contribution

Adeodatus Yuda Handaya conceived the study and approved the final draft. Aditya Rifqi Fauzi drafted the manuscript. Ahmad Shafa Hanif, Joshua Andrew, Kevin Radinal, and Azriel Farrel Kresna Aditya critically revised the manuscript for important intellectual content. Adeodatus Yuda Handaya, Aditya Rifqi Fauzi, Ahmad Shafa Hanif, Joshua Andrew, Kevin Radinal, and Azriel Farrel Kresna Aditya facilitated all project-related tasks.

## Registration of research studies

The manuscript is a case report, not considered a formal research involving participants.

## Guarantor

Adeodatus Yuda Handaya.

## Declaration of competing interest

No potential conflict of interest relevant to this article was reported.
